# Microtubule regulators act in the nervous system to modulate fat metabolism and longevity through DAF‐16 in *C. elegans*

**DOI:** 10.1111/acel.12884

**Published:** 2019-01-14

**Authors:** Aiping Xu, Zhao Zhang, Su‐Hyuk Ko, Alfred L. Fisher, Zhijie Liu, Lizhen Chen

**Affiliations:** ^1^ Barshop Institute for Longevity and Aging Studies San Antonio Texas; ^2^ Department of Cell Systems and Anatomy UTHSCSA San Antonio Texas; ^3^ Department of Molecular Medicine UTHSCSA San Antonio Texas; ^4^ Center for Healthy Aging UTHSCSA San Antonio Texas; ^5^ Division of Geriatrics, Gerontology, and Palliative Medicine, Department of Medicine UTHSCSA San Antonio Texas; ^6^ GRECC, South Texas VA Healthcare System San Antonio Texas; ^7^ Present address: Division of Geriatrics and Gerontology, Department of Medicine University of Nebraska Medical Center Omaha Nebraska

**Keywords:** *daf‐16*, *efa‐6*, fat metabolism, longevity, microtubules, neuronal aging

## Abstract

Microtubule (MT) regulation is involved in both neuronal function and the maintenance of neuronal structure, and MT dysregulation appears to be a general downstream indicator and effector of age‐related neurodegeneration. But the role of MTs in natural aging is largely unknown. Here, we demonstrate a role of MT regulators in regulating longevity. We find that loss of EFA‐6, a modulator of MT dynamics, can delay both neuronal aging and extend the lifespan of *C. elegans*. Through the use of genetic mutants affecting other MT‐regulating genes in *C. elegans*, we find that loss of MT stabilizing genes (including *ptrn‐1* and *ptl‐1*) shortens lifespan, while loss of MT destabilizing gene *hdac‐6* extends lifespan. Via the use of tissue‐specific transgenes, we further show that these MT regulators can act in the nervous system to modulate lifespan. Through RNA‐seq analyses, we found that genes involved in lipid metabolism were differentially expressed in MT regulator mutants, and via the use of Nile Red and Oil Red O staining, we show that the MT regulator mutants have altered fat storage. We further find that the increased fat storage and extended lifespan of the long‐lived MT regulator mutants are dependent on the DAF‐16/FOXO transcription factor. Our results suggest that neuronal MT status might affect organismal aging through DAF‐16‐regulated changes in fat metabolism, and therefore, MT‐based therapies might represent a novel intervention to promote healthy aging.

## 1 INTRODUCTION

Microtubules (MTs) are essential cytoskeletal structures for most eukaryotic cells and MT regulators have been implicated in many human diseases. MTs are polymeric hollow rods built from dimers of α‐ and β‐tubulin that are arranged in a head‐to‐tail manner. MTs are highly dynamic structures that constantly undergo growth and shrinkage/catastrophe (Mitchison & Kirschner, 1984). MT dynamics and stability can be modulated by a large number of MT‐interacting proteins. Some MT‐associated proteins (MAPs), such as Tau, bind along the MT structure to promote MT assembly and stability by protecting MTs from severing by destabilizing factors (Kapitein & Hoogenraad, 2015). Due to the larger cytoplasmic volume and polarized morphology, neurons rely more on MT function compared with other cell types. MTs serve as tracks for long‐distance transport and play a critical role in the establishment and maintenance of neuronal structure and polarity (Kapitein & Hoogenraad, 2015). Consequently, abnormalities in MT organization and dynamics, as well as MT protein expression or distribution, have been observed in many neurological disorders. Mutations in tubulin genes or MAPs have also been reported to affect neuronal integrity during aging (Chew, Fan, Gotz, & Nicholas, 2013; Pan, Peng, Chen, & McIntire, 2011). The importance of MT regulation in healthy neuronal aging is underscored by the critical role of Tau in MT stabilization and its dysfunction related to neurodegenerative diseases where the impairment of axonal transport is a common factor (Baird & Bennett, 2013).

The nervous system is the central integrator of information regarding the internal milieu of an organism and the external environment, and also plays a key role in regulating aging and longevity (Alcedo, Flatt, & Pasyukova, 2013). It allows animals to process and transmit extrinsic signals to neuronal or non‐neuronal endocrine cells that regulate the release of hormones involved in growth and metabolism. In addition, studies have shown that chemosensory neurons regulate animal longevity in *C. elegans*. The laser ablation of gustatory neurons or olfactory neurons is sufficient to extend lifespan, and these two types of neurons function in parallel in regulating lifespan (Alcedo & Kenyon, 2004). Mutations that disrupt the sensory cilia used by these neurons also lead to defective sensory perception and increased longevity (Apfeld & Kenyon, 1999). Similarly, the loss of olfactory function in *Drosophila* has also been shown to increase lifespan (Libert et al., 2007). The neuronal influence on lifespan is mediated by several mechanisms including alterations in the *daf‐2*/insulin/insulin‐like growth factor‐1 signaling (IIS) pathway (Alcedo & Kenyon, 2004; Apfeld & Kenyon, 1999). This pathway plays an evolutionarily conserved role in regulating longevity, stress resistance, and metabolism in various model organisms (Partridge, Alic, Bjedov, & Piper, 2011; Taguchi & White, 2008; Tatar, Bartke, & Antebi, 2003).

Given the critical role of MTs in regulating neuronal function and key role of the nervous system in modulating aging, we tested the hypothesis that MT regulators can affect neuronal function to thereby alter organismal aging. MT regulator mutants have been implicated in the aging process, but they have not been specifically studied and the underlying mechanisms are unknown. For example, loss of PTL‐1, the *C.elegans *ortholog of Tau, has been shown to accelerate neuronal aging and shorten animal lifespan (Chew et al., 2013). Loss of MT regulator *Stathmin* resulted in reduced levels of stable axonal MTs and shortened lifespan in *Drosophila* (Duncan, Lytle, Zuniga, & Goldstein, 2013). In this study, we demonstrate that loss of EFA‐6, a negative regulator of MT growth, delays neuronal aging and extends organismal lifespan and health span in *C. elegans*. Mutations in other MT regulator genes, including *hdac‐6* and *ptrn‐1*, can also affect lifespan. We show by tissue‐specific transgene rescue that these MT regulators function in the nervous system to modulate longevity. Through the use of RNA‐seq analysis, we show that genes involved in lipid metabolism are differentially expressed in MT regulator mutants and lead to an increase in fat storage. Lastly, via epistasis experiments we show that the lifespan extension phenotypes of the *efa‐6* and *hdac‐6* mutants depend on the DAF‐16/FOXO transcription factor. Our results suggest that neuronal MT status can affect organismal longevity through modulating fat metabolism.

## 2 RESULTS

### 2.1 Loss of EFA‐6 delays neuronal aging and extends lifespan

**Figure 1:**
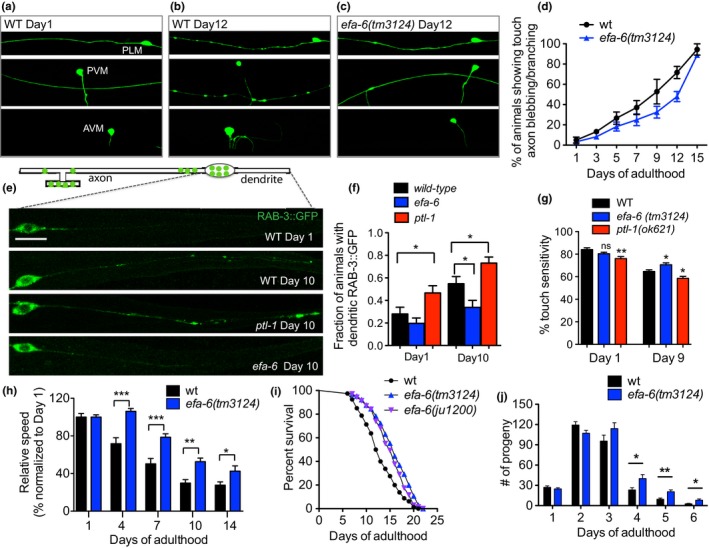
Loss of EFA‐6 delays neuronal aging and extends lifespan and health span. (a) Representative images of touch sensory neurons in wild‐type Day 1 young adults. Neurons are visualized using *Pmec‐7::GFP* reporter (*muIs32*). (b–c) Representative images of touch sensory neurons in wild‐type and *efa‐6(tm3124)* Day 12 aged animals. The top two panels show blebbing neuron processes and the bottom panel shows ectopic branches of AVM. (d) Percentage of animals showing branching and/or blebbing touch neurons in wild‐type and *efa‐6(tm3124)*. Values are mean percentage from three experiments, each experiment started with 20 worms. ***p = 0.0009, two‐way ANOVA. (e) Representative images showing the localization of synaptic vesicles labeled with RAB‐3::GFP (*jsIs821*) in the dendritic process of PLM. In aged neurons, there are more vesicles ectopically localized in dendrite, and this age‐dependent mis‐localization is enhanced in *ptl‐1* mutants but partially rescued in *efa‐6* mutants. (f) Fraction of the animals showing dendritic RAB‐3::GFP signals. *N *≥ 60, one‐way ANOVA. (g) Percentage of positive touch response per animal of indicated genotypes at Day 1 and Day 9. *N* = 30, one‐way ANOVA. (h) Motility of wild‐type and *efa‐6(tm3124) *mutant animals at different stages is assessed via the measurement of thrashing behavior in liquid. The values represent the normalized average speed of body bends during a 30‐s period. *N* ≥ 12 for each bar. Student's *t* test. (i) Survival curve for wild‐type and two independent *efa‐6* deletion mutants. ***p < 0.0001 for *wt *vs *tm3124,* **p = 0.0032 for *wt *vs *ju1200*. Log‐rank test. (j) Number of progenies per 24 hr from wild‐type and *efa‐6(tm3124) *mutants at different stages. *N* ≥ 12 for each bar. Student's *t* test

We have previously identified EFA‐6 as a modulator of neuronal MT dynamics (Chen et al., 2015). MTs are crucial cytoskeleton for neuronal integrity and MT defects have been characterized in different neurodegenerative conditions (Dubey, Ratnakaran, & Koushika, 2015). As a result, we tested whether EFA‐6 plays a role in maintaining neuronal integrity during aging. Mechanosensory neurons (touch neurons) offer an excellent model to study changes associated with neuronal aging in *C. elegans *(Toth et al., 2012). We used a *mec‐7p::*GFP reporter to label mechanosensory neurons and then examined the effect of age on their structure. During aging, these axons of these neurons accumulated branches and blebs, and cell bodies displayed branching as the animals aged. (Figure 1a–d). We found that these age‐dependent morphological changes were significantly delayed in *efa‐6(tm3124)* mutants. By Day 9 of adulthood, more than 50% of the wild‐type animals showed blebbing/branching phenotype, whereas only around 30% of *efa‐6* mutants at Day 9 displayed this defect. By Day 12, the proportion of wild‐type animals with abnormal touch neuron morphology reached 70%, compared to less than 50% in *efa‐6* mutants at this stage (Figure 1d). This delayed neuronal aging in *efa‐6* mutants is contrary to the previously reported, accelerated neuronal aging phenotype of the *ptl‐1* mutants, which in contrast to *efa‐6 *mutations results in the destabilization of the MTs (Chew et al., 2013).

MT‐based transport is essential for neuronal function, and the impairment of this process is a common factor in several neurodegenerative diseases (Franker & Hoogenraad, 2013). To test whether loss of EFA‐6 affects intracellular transport, we examined the distribution of synaptic vesicles that depend on MT‐based transport. Aging wild‐type animals displayed an increase in the ectopic accumulation of synaptic vesicles labeled by RAB‐3::GFP in the dendritic region of the PLM neuron (Figure 1e–f). This age‐dependent change in the dendritic distribution of RAB‐3::GFP was partially rescued in *efa‐6* mutants but exaggerated in *ptl‐1* mutants (Figure 1e–f). Sensitivity to light touches, a function of mechanosensory neurons, decreases in aged wild‐type animals (Jiang et al., 2015). Consistent with the age‐associated morphological changes and ectopic RAB‐3 distribution, we found that the touch sensitivity was better maintained in aged *efa‐6* mutants but further diminished in aged *ptl‐1* mutants (Figure 1g).

As worms age, their mobility declines, and the correlation between age‐dependent decreases in mobility and defects in neuronal morphology has been previously reported (Tank, Rodgers, & Kenyon, 2011). Since loss of EFA‐6 delayed neuronal aging, we asked whether it affected age‐dependent mobility decline. We measured the speed of the animal bending in liquid during aging. We found that the movement speed of the animals gradually declined with age in the wild‐type animals. The *efa‐6(tm3124)* mutants displayed significantly higher movement speed at all stages examined (Supporting information Figure S1a), and they showed a slower rate of reduction in the relative speed (normalized to Day 1) (Figure 1h). Overall, these results suggest that the loss of EFA‐6, a regulator of MT dynamics, could delay age‐dependent changes in neuronal morphology and neuronal function that would lead to improved mobility during aging.

Given the involvement of EFA‐6 in neuronal age‐related phenotypes, we examined the lifespan of the *efa‐6* mutants. We found *efa‐6(tm3124)* had a longer lifespan compared to wild‐type controls, with a more obvious increase in mean lifespan than that in maximal lifespan (Figure 1i). When we performed the lifespan assays using NGM plates that do not contain FUDR, we noted that *efa‐6(tm3124)* mutants continued to lay eggs until later in adulthood, when the wild‐type animals had already stopped producing progeny. We then evaluated this extended reproduction period by measuring the number of progenies produced by wild‐type and *efa‐6 *mutant worms from Days 1 through 6 of adulthood. We found that the *efa‐6* mutants had slightly fewer progeny on Day 1 and Day 2, but after Day 3, the *efa‐6* mutants produced more progeny compared to wild‐type worms of the same age, with total progeny number similar to wild‐type (Figure 1j, Supporting information Figure S1b‐c).

### 2.2 EFA‐6 functions in neurons to regulate organismal longevity

**Figure 2:**
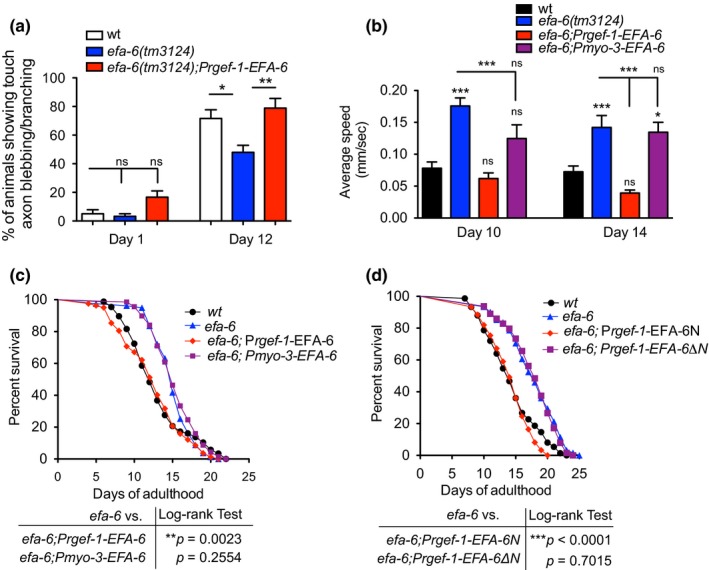
EFA‐6 acts in neurons and requires its *N*‐terminus to regulate aging. (a) Delayed touch neuronal aging in *efa‐6* mutants can be rescued by transgenic expression of EFA‐6 in neurons. Percentage of animals showing branching and/or blebbing touch neurons in wild‐type, *efa‐6(tm3124) *and *efa‐6(tm3124);Prgef‐1‐EFA‐6*. Values are mean percentage from three experiments. Each experiment started with 20 worms. One‐way ANOVA. (b) Mobility phenotype in *efa‐6* mutants can be rescued by transgenic expression of EFA‐6 in neurons. The values represent the average speed of body bends during a 30‐s period. *N* ≥ 12 for each bar. *N *≥ 10 for each bar. One‐way ANOVA. (c) Survival curves for indicated genotypes. Neuronal expression (P*regf‐1*), but not muscular expression (P*myo‐3*), of EFA‐6 is able to rescue the lifespan extension phenotype in *efa‐6(tm3124)* mutants. (d) Survival curves for indicated genotypes. The N‐terminus of EFA‐6 is required for its function. Transgenic expression of the N‐terminus, but not a truncated EFA‐6 lacking N‐terminus, rescues lifespan phenotype in *efa‐6(tm3124)* mutants

We next sought to confirm that the effects observed in our *efa‐6(tm3124)* worms were due to the loss of function of *efa‐6* via the use of tissue‐specific transgenes expressing *eaf‐6*. While high level expression of *efa‐6 *in the nervous system can disrupt development (Chen et al., 2015), lower level of expression did revert the *efa‐6 *neuronal aging phenotype back to wild‐type without causing developmental defects (Figure 2a). We also found that neuronal expression of *efa‐6 *was able to completely rescue the enhanced mobility of the *efa‐6* mutants during aging (on both Day 10 and Day 14), whereas muscle‐specific expression of *efa‐6 *had no effect (Figure 2b). Remarkably, neuronal expression of *efa‐6 *was able to completely rescue the lifespan phenotype of *efa‐6(tm3124) *mutant. In contrast, a transgene expressing *efa‐6 *only in the muscle failed to rescue the lifespan phenotype (Figure 2c). We further tested tissue‐specific EFA‐6 expression in intestine (*Pges‐1*), epidermis (*Pdpy‐7*), and germline (*Ppie‐1*) for the effect on lifespan extension at *efa‐6* mutant background. The germline and epidermis‐specific transgenes were not able to rescue the lifespan phenotype of *efa‐6, *while the intestinal transgenic expression of EFA‐6 was able to modestly rescue (Supporting information Figure S2). These data suggest that EFA‐6 can act in neurons to modulate organismal aging.

The EFA‐6 protein is dependent on its N‐terminus to regulate cortical MT growth and axon regeneration (Chen et al., 2011; O'Rourke, Christensen, & Bowerman, 2010). We next tested whether the N‐terminus of the EFA‐6 protein is also required for its role in longevity. The neuronal expression of a 150 amino acid fragment from the N‐terminus, but not the EFA‐6 protein lacking the N‐terminus (EFA‐6∆N), could reduce the increased lifespan of the *efa‐6 *mutant (Figure 2d). Together these results suggest that the N‐terminus is necessary for the effects of *efa‐6* on longevity.

### 2.3 Mutations in microtubule regulators affect longevity

**Figure 3:**
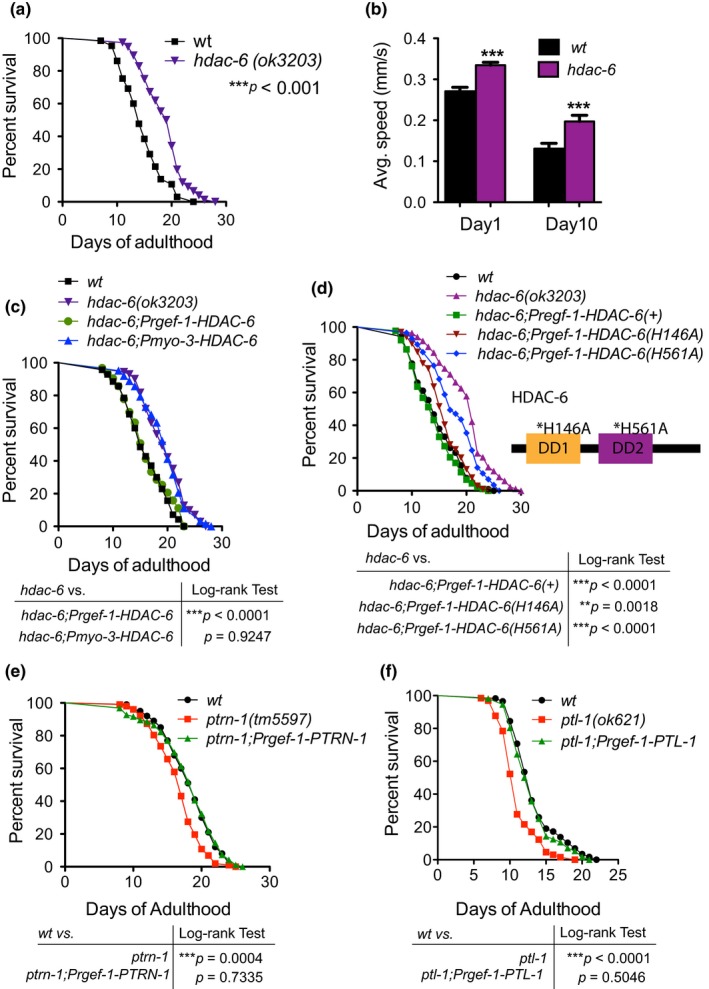
Microtubule regulators function in the nervous system to regulate lifespan. (a) Survival curve for *hdac‐6(ok3203)* and control. Loss of HDAC‐6 extends lifespan. Log‐rank test. (b) *hdac‐6(ok3203) *mutant animals display enhanced mobility. The values represent the average speed of body bends at adult Day 1 and Day 10 stages. (c) Survival curves for indicated genotypes. Neuronal expression (*regf‐1* promoter), but not muscular expression (*myo‐3* promoter) of HDAC‐6, is able to rescue the lifespan extension phenotype in *hdac‐6(ok3203)* mutants. (d) Schematic structure of HDAC‐6 protein and survival curves. Lifespan extension in *hdac‐6(ok3203)* can be fully rescued by neuronal expression of a wild‐type HDAC‐6 protein, but only partially rescued by mutant forms of HDAC‐6. Mutant HDAC‐6 with a mutation in DD2 (tubulin deacetylase domain) has a weaker rescuing effect compared to DD1. (e) Survival curves for indicated genotypes. Loss of PTRN‐1 shortens lifespan and neuronal transgenic expression of PTRN‐1 fully rescues the phenotype. (f) Survival curves for wild‐type, *ptl‐1(ok621)* and *ptl‐1;Prgef‐1‐PTL‐1*. Lifespan reduction in *ptl‐1(ok621)* is fully rescued by transgenic expression of PTL‐1 in the nervous system

We then tested whether *efa‐6 *was unique or whether other MT regulators played similar roles in modulating longevity. We first tested the *hdac‐6(ok3203)* mutant, which affects a histone deacetylase that also targets nonhistone proteins, including α‐tubulin (Li, Jiang, Chang, Xie, & Hu, 2011). Specifically, HDAC6 deacetylates α‐tubulin to reduce MT stability (Matsuyama et al., 2002). We therefore tested the lifespan of *hdac‐6* mutant animals containing a deletion mutation. We observed extended lifespan and enhanced mobility in *hdac‐6(ok3203*) mutant, similar to *efa‐6* mutants (Figure 3a,b). To ask whether *hdac‐6* acts in the nervous system to modulate longevity, we expressed HDAC‐6 using pan‐neuronal promoter in the *hdac‐6(ok3203)* background. We found that neuronal expression of HDAC‐6 could completely revert the extended lifespan to a wild‐type level (Figure 3c), indicating that *hdac‐6* also acts within neurons. Inhibiting HDACs is known to extend lifespan in *Drosophila* and *C. elegans* through epigenetic effects (Pasyukova & Vaiserman, 2017), although a role for HDAC6 in aging has not been reported previously.

HDAC6 belongs to the class IIb family of HDACs, which is predominantly localized in the cytosol and prefers nonhistone targets (Simoes‐Pires et al., 2013). A unique feature of HDAC6 is the presence of two catalytic domains. The second deacetylase domain (DD2) has been demonstrated to deacetylate tubulin specifically (Haggarty, Koeller, Wong, Grozinger, & Schreiber, 2003; Kaluza et al., 2011). Loss of DD2 activity in HDAC6 is sufficient to rescue the MT defects induced by ectopic human tau expression in Drosophila (Xiong et al., 2013). To test whether the tubulin‐specific deacetylase activity is responsible for the role of HDAC‐6 in regulating longevity, we generated transgenic animals with pan‐neuronal expression of HDAC‐6 (H146A) or HDAC‐6 (H561A), two mutant forms with the conserved catalytic residue in DD1 and DD2 mutated. We found that both mutant HDAC‐6 proteins were able to partially rescue the lifespan phenotype in *hdac‐6* mutant, but DD2 mutant had a weaker rescuing effect (Figure 3d). These data indicate that tubulin‐specific deacetylase activity conferred by DD2 is perhaps more critical for the role of HDAC‐6 in regulating lifespan.

We further extended our study to more MT regulator genes including *ptrn‐1 *and *ptl‐1*. The CAMSAP family of proteins are a group of conserved, MT minus end‐binding proteins. PTRN‐1, the CAMSAP homolog in *C. elegans*, promotes MT stability in neurons and epidermis (Marcette, Chen, & Nonet, 2014; Richardson et al., 2014; Wang et al., 2015). *ptrn‐1* mutants showed shortened lifespan and the difference between control and *ptrn‐1* was moderate but significant. Similar to *efa‐6* and *hdac‐6* mutants, the lifespan phenotype in *ptrn‐1* mutant could be rescued by the transgenic expression of *ptrn‐1* in neurons (Figure 3e). PTL‐1 has been previously reported to regulate both neuronal and organismal aging (Chew et al., 2013). Consistent with the previous studies, we found that *ptl‐1(ok621)* null mutants displayed shortened lifespan, which can be rescued by pan‐neuronal expression of PTL‐1 (Figure 3f), further supporting that neuronal MT regulation can affect longevity.

### 2.4 Aberrant expression of genes involved in fat metabolism and altered fat storage in microtubule regulator mutants

**Figure 4:**
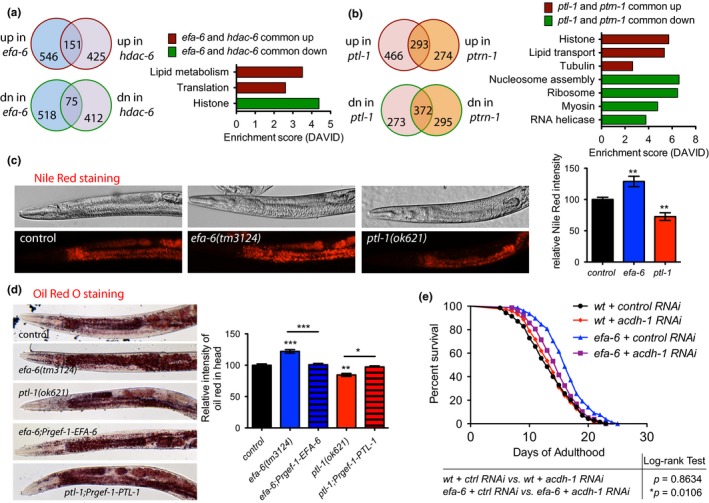
MT regulator mutants show aberrant expression of lipid metabolism‐related genes and altered fat storage. (a) RNA‐seq reveals differentially expressed genes in MT regulator mutants. Venn diagram showing the overlapping genes that are up‐regulated or down‐regulated in *efa‐6(tm3124)* and *hdac‐6(ok3203)* mutants. Significant gene ontology (GO) cluster terms from the shared up‐ and down‐regulated genes. Lipid metabolism cluster is the most significantly enriched cluster in the 151 shared up‐regulated genes. (b) Venn diagram showing the overlapping genes that are up‐regulated or down‐regulated in *ptl‐1(ok621)* and *ptrn‐1(tm5597)* mutants. Significant gene ontology (GO) cluster terms from the shared up‐ and down‐regulated genes. (c) Representative images and quantification of fixed Nile Red staining of indicated genotypes at Day 1 young adult stage. A bright field image of the same animal is also presented. Nile Red staining is increased in *efa‐6* mutants and reduced in *ptl‐1* mutants. (d) Representative images and quantification of fixed Oil Red O staining of indicated genotypes at Day 1 young adult stage. *N *≥ 10, one‐way ANOVA. (e) Survival curves of indicated strains. *acdh‐1* RNAi partially suppressed the lifespan phenotype in *efa‐6* mutants

To understand how the MT regulator mutants might affect animal aging, we performed RNA‐seq to identify genes that are differentially regulated in four of these mutants, including the long‐lived *efa‐6(tm3124)* and *hdac‐6(ok3203)*, and the short‐lived *ptl‐1(ok621)* and *ptrn‐1(tm5597) *mutants. We compared the transcriptome of each of these mutants with that of wild‐type control and identified several hundreds of up‐ and down‐regulated genes with the threshold set at ***p < 0.001 (Figure 4a, b, Supporting information Table S1). To narrow down the potential effector genes, we focused on the overlapped genes between the two long‐lived MT mutants (*efa‐6* and *hdac‐6*), as well as those between the two short‐lived MT mutants (*ptl‐1* and *ptrn‐1*). We then used the DAVID functional annotation program to identify biologic themes within the common up‐ and down‐regulated genes. Within the common up‐regulated genes in *efa‐6* and *hdac‐6* mutants, we found that genes involved in lipid metabolism are significantly enriched (Figure 4a,b and Supporting information Table S1). We also found that histone genes are enriched in the common down‐regulated genes between *efa‐6* and *hdac‐6* mutants, as well as in the common up‐regulated genes in *ptl‐1* and *ptrn‐1* mutants. Multiple histone modification has been linked to aging regulation through epigenetic mechanisms (Tessarz et al., 2014). Elevated histone expression has been shown to extend lifespan in yeast, and RNAi of *C. elegans* histone genes has been reported to extend the lifespan of *daf‐2;aak‐1;aak‐2* dauer larvae (Xie & Roy, 2012), but the effects of the changes in histone gene expression on aging of multicellular organisms are currently unclear.

We next looked into the genes whose expression is oppositely affected between the two long‐lived (*efa‐6* and *hdac‐6*) and the two short‐lived (*ptl‐1* and *ptrn‐1*) mutants (Supporting information Figure S3). Among them, *nap‐1* and *acdh‐1* have been previously linked to lipid metabolism and aging regulation. NAP‐1 is a conserved nucleosome assembly protein (NAP) family member that can function as a sensor of cholesterol and interact with DAF‐16 to regulate transcription of longevity genes (Cheong et al., 2013; Ihara, Uno, Miyatake, Honjoh, & Nishida, 2017). NAP‐1 overexpression is sufficient to extend lifespan and enhance fat storage in wild‐type animals (Cheong et al., 2013). *acdh‐1 *encodes a short‐chain acyl‐CoA dehydrogenase that catalyzes fatty acid beta‐oxidation. ACDH‐1 protein level is up‐regulated in *eat‐2* mutants, and *acdh‐1* RNAi significantly decreases the lifespan of *eat‐2* (Yuan et al., 2012).

Given that genes involved in fat metabolism were differentially expressed in MT mutants, we tested whether fat metabolism was affected in these mutants. We compared fat staining in fixed wild‐type and mutant animals using the lipophylic dye Nile Red, which stains neutral lipids in fixed tissues (Brooks & Liang, 2009). We found that the intensity of stained lipid droplets is significantly reduced in *ptl‐1*, but increased in *efa‐6* mutants (Figure 4c), correlating with the changes in lifespan of these mutants. We also confirmed the fat staining phenotype using Oil Red O staining (Figure 4d), which stains also neutral lipids such as triglycerides (O'Rourke, Soukas, Carr, & Ruvkun, 2009). To determine whether EFA‐6 and PTL‐1 act in the nervous system to regulate fat accumulation, we performed transgenic rescue experiments. We found that the fat storage phenotype in *efa‐6* and *ptl‐1* mutants could be rescued by neuron‐specific transgenic expression of EFA‐6 and PTL‐1, respectively (Figure 4c). Emerging studies from both invertebrates and mammals suggest that elevated fat storage can affect longevity (Hansen, Flatt, & Aguilaniu, 2013; Steinbaugh et al., 2015). To test whether lipid metabolism is critical for the role of MT regulators in modulating longevity, we treated *efa‐6* mutants with *acdh‐1* RNAi. *acdh‐1* RNAi partially suppressed the lifespan extension in efa‐6 mutants, but did not affect lifespan at wild‐type background (Figure 4e). However, *acdh‐1* RNAi did not affect the fat accumulation phenotype of the *efa‐6* mutants (Supporting information Figure S3c), this could indicate that alterations in both lipid production and degradation might be more important than a shift toward lipid storage in mediating the effects of MT regulators on aging. Taken together, these data suggest that MT regulators might act in neurons to modulate aging through alterations in lipid metabolism.

### 2.5 The roles of microtubule regulators in modulating aging and metabolism require DAF‐16

**Figure 5:**
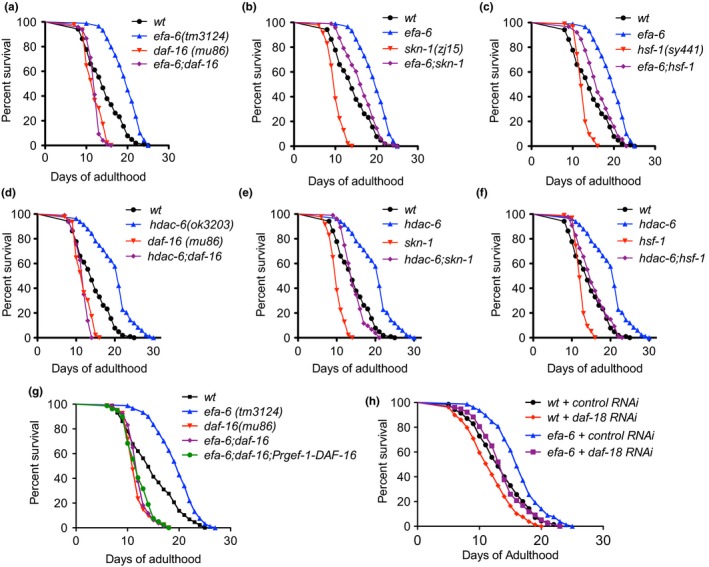
The aging effects in microtubule regulators mutants require *daf‐16* activity. (a) The lifespan extension phenotype in *efa‐6* mutants was suppressed by *daf‐16* mutation. *efa‐6;daf‐16* double mutant resembles *daf‐16* single mutant, suggesting that *efa‐6* phenotype is dependent on *daf‐16*. (b–c) Additive effect between *efa‐6* and *skn‐1* (or *hsf‐1*) on lifespan suggests that they function in parallel. (d–f) The lifespan extension phenotype in *hdac‐6* mutants was also suppressed by *daf‐16* mutation, but not *skn‐1* or *hsf‐1* mutation. (g) Re‐expressing DAF‐16 in the nervous system was not sufficient to rescue the shortened lifespan in *efa‐6;daf‐16* double mutants. *efa‐6;daf‐16;Prgef‐1‐DAF‐16* animals were generated by co‐injecting Prgef‐1‐DAF‐16A and Prgef‐1‐DAF‐16F plasmids to *efa‐6;daf‐16* strain. (h) *daf‐18* RNAi and *efa‐6(tm3124)* displayed an additive effect on lifespan

We next investigated the interactions of MT regulator genes with other aging‐regulatory pathways. DAF‐16/FOXO, HSF‐1, and SKN‐1/NRF2 are the three of the most important lifespan‐modulatory transcription factors that have been identified so far (Murphy & Hu, 2013). We generated double mutants of MT regulator genes (*efa‐6* or *hdac‐6*) and aging regulators (*daf‐16, hsf‐1, or skn‐1*) and compared the lifespan phenotype in double mutants with each single mutant. The lifespan extension phenotype induced by loss of EFA‐6 or HDAC‐6 was completely abolished in the absence of DAF‐16, but was maintained, though somewhat reduced, in the *hsf‐1* and *skn‐1* mutants (Figure 5a–f), suggesting that *efa‐6* and *hdac‐6* regulate longevity in a DAF‐16‐dependent manner. We then asked whether the differentially expressed genes in the MT regulator mutants identified in the RNA‐seq overlap with DAF‐16 targets. We compared the list of differentially expressed genes with the list of known DAF‐16 targets (Tepper et al., 2013). The strongest overlapping was found in the group of up‐regulated genes in efa‐6 mutants, with 81 (11.6%) direct and 137 (19.7%) indirect targets of DAF‐16 out of 697 genes (Supporting information Table S1), supporting the notion that the role of MT regulators in modulating aging is mediated by DAF‐16.

Our data described above suggested that MT regulators predominantly function in the nervous system to modulate lifespan, thus we tested whether neuronal DAF‐16 alone was sufficient to mediate the role of MT regulators on modulating lifespan. Re‐expressing DAF‐16 in the nervous system was not able to rescue the shortened lifespan in *efa‐6;daf‐16* double mutants (Figure 5g), suggesting that DAF‐16 is required in a cell‐nonautonomous manner for MT regulators to modulate longevity.

IIS within the nervous system plays an important role in longevity regulation in both *C. elegans* and mouse (Iser, Gami, & Wolkow, 2007; Taguchi, Wartschow, & White, 2007; Wolkow, Kimura, Lee, & Ruvkun, 2000), and DAF‐16/FOXO is one of the most important downstream transcription factors in IIS pathway. We then asked whether MT regulators modulate lifespan through the IIS pathway. IIS is negatively regulated by the lipid phosphatase DAF‐18, the ortholog of the human tumor suppressor PTEN, and *daf‐18* RNAi has been shown to increase the activity of the IIS pathway (Gil, Malone Link, Liu, Johnson, & Lees, 1999; Mihaylova, Borland, Manjarrez, Stern, & Sun, 1999; Ogg & Ruvkun, 1998). We found that *daf‐18* RNAi significantly shortened the lifespan and displayed additive effect with *efa‐6(tm3124)* (Figure 5h), suggesting that *efa‐6* might not function directly through IIS pathway.

**Figure 6:**
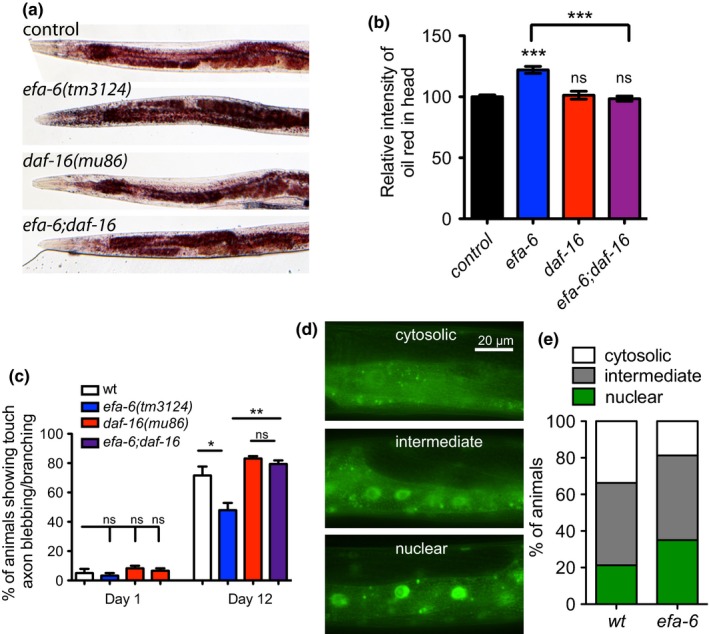
The role of *efa‐6* is DAF‐16‐dependent. (a–b) Fat storage phenotype in *efa‐6* mutants also depends on DAF‐16. Representative images and quantification of fixed Oil Red O staining of indicated genotypes at Day 1 young adult stage. *N* ≥ 10, one‐way ANOVA. (c) The neuronal aging protection effect of *efa‐6* mutants was abolished by *daf‐16* mutation. Percentage of animals showing branching and/or blebbing touch neurons in wild‐type, *efa‐6(tm3124) *and *efa‐6;daf‐16 *animals. Values are mean percentage from three experiments. Each experiment started with 20 worms. One‐way ANOVA. (d–e) Representative images and quantification of DAF‐16::GFP (*muIs71*) at L4 stage. *N* = 80 for each genotype. Chi‐square test, *p = 0.0466

As FOXO transcription factors play a conserved role in regulating energy homeostasis and lipid metabolism, we tested whether the fat storage effect in MT regulator mutants was dependent on DAF‐16. We found that the increase of fat storage in *efa‐6* mutants was significantly attenuated in the *efa‐6;daf‐16* mutants (Figure 6a–b). During *C. elegans* aging, touch sensory neurons display morphological changes, and *daf‐16* is required to maintain touch neuron integrity (Pan et al., 2011; Tank et al., 2011; Toth et al., 2012). We tested whether the delayed neuronal aging in *efa‐6* mutants was dependent on DAF‐16. We found that *daf‐16* mutants showed mild defects in touch neuron morphology compared to wild‐type control on both Day 1 and Day 12 of adult life (Figure 6c). Loss of DAF‐16 abolished the protective effect of *efa‐6(tm3124)* on adult Day 12 (Figure 6c). Taken together, these data suggest that MT regulators influence neuronal aging and fat storage in a *daf‐16*‐dependent manner.

The activity of DAF‐16/FOXO is regulated by a broad variety of stimuli that control post‐translational modification, subcellular localization, DNA binding, and transcriptional activity. The nuclear accumulation of DAF‐16 is required for the induction of its target genes, and nuclear enrichment of DAF‐16::GFP is observed in many long‐lived mutants. We then evaluated whether DAF‐16 nuclear localization was affected in *efa‐6* mutants using an integrated DAF‐16::GFP transgenic line. We found an increase in the population of animals with strong nuclear localization (Figure 6d–e), further supporting the DAF‐16‐dependent function of MT regulators.

## 3 DISCUSSION

### 3.1 Microtubule‐associated proteins as novel longevity regulators

Microtubule dysfunction is associated with age‐related neurodegenerative diseases (Dubey et al., 2015). Microtubule regulators have been implicated, but not been directly linked to lifespan modulation. Recently, studies on PTL‐1, the worm homolog of mammalian Tau protein, have demonstrated that PTL‐1 is involved in age‐related preservation of neuronal structural integrity and in promoting a normal lifespan (Chew et al., 2013). Similarly, a previous study in *Drosophila* showed that loss of stathmin, a microtubule regulator, led to a reduction in axonal MT stability and a decrease in lifespan (Duncan et al., 2013). These studies suggested that a disruption in neuronal MTs might be detrimental for longevity. However, whether manipulating MTs can promote longevity remains unknown.

Here, we demonstrate that EFA‐6 normally negatively regulates neuronal integrity and synaptic vesicle distribution during aging, while in contrast PTL‐1 normally protects neuronal structural integrity and function. These neuronal effects are linked to the influence of *efa‐6 and ptl‐1 *on organismal longevity. Loss of EFA‐6 extends lifespan and the lifespan extension phenotype can be completely rescued by neuronal expression of EFA‐6. To test whether the impact on longevity is general property of MT regulators, we further tested a set of genetic mutations of microtubule regulator genes and identified *hdac‐6* and *ptrn‐1* as longevity regulating genes. Similar to EFA‐6 and PTL‐1, these MT regulators appear to act in the nervous system to control lifespan, as pan‐neuronal expression of these genes is sufficient to rescue the lifespan phenotype in the mutants. Previous genome‐wide RNAi screens for genes involved in lifespan regulation have not identified these microtubule regulators, possibly because most *C. elegans* neurons are resistant to RNAi feeding (Asikainen, Vartiainen, Lakso, Nass, & Wong, 2005).

We found that *efa‐6* and *hdac‐6* mutants are long‐lived, while *ptl‐1and ptrn‐1* mutants are short‐lived. The opposing phenotypes in the two groups of mutants suggest a connection between their effects on MTs and the resulting effect on aging. Specifically, PTL‐1 and PTRN‐1 have been shown to promote MT stability. PTL‐1 is highly conserved in both structure and function to *Tau*, which binds to and stabilizes MTs (Drubin & Kirschner, 1986). PTRN‐1 promotes MT stability in *C. elegans* neurons and epidermis (Marcette et al., 2014; Richardson et al., 2014; Wang et al., 2015). On the other hand, EFA‐6 appears to promote MT catastrophe (O'Rourke et al., 2010), and HDAC6 deacetylates alpha‐tubulin and negatively regulates MT stability (Matsuyama et al., 2002). Hence, it appears that increased MT stability leads to increased organismal longevity whereas decreased stability shortens lifespan. In people, reduced MT stability is associated with several neurodegenerative diseases such as Alzheimer's disease and Parkinson's disease. However, hyperstable MTs can also lead to neurodegeneration as found in Hereditary Spastic Paraplegia (Dubey et al., 2015). Therefore, the right level of MT stability might be critical for neuronal function. Perturbing the balance in MT dynamics might disrupt neuronal function, while promoting the homeostasis is protective for neurons to preserve function during aging. Further investigation on how those MT regulators impact MT dynamics will be needed to understand their roles influencing the aging process.

### 3.2 MT regulators act in the nervous system to influence organismal longevity

The nervous system is known to modulate lifespan in diverse species. It detects sensory cues from the environment and internal signals from the animal, and coordinates organismal metabolic homeostasis and energy balance (Riera & Dillin, 2016). Previous studies have shown that chemosensory neurons regulate animal longevity in *C. elegans* (Alcedo & Kenyon, 2004). Mutations that disrupt sensory cilia lead to defective sensory perception and promote longevity (Apfeld & Kenyon, 1999). Similarly, loss of olfactory function in *Drosophila* has also been shown to increase lifespan (Libert et al., 2007). Later studies have then shown that these sensory neurons influence longevity through multiple mechanisms. First, specific sensory neurons synthesize INS‐6 and DAF‐28, which act via DAF‐2 to inhibit the accumulation of DAF‐16 in the nucleus and the subsequent activation of DAF‐16 target genes (Kenyon, 2010; Riera, Merkwirth, Magalhaes Filho, & Dillin, 2016). Second, lifespan extension conferred by dietary restriction has been shown to be mediated by SKN‐1 that acts in the ASI sensory neurons (Bishop & Guarente, 2007). Finally, the AFD thermosensory neurons promote lifespan at warm temperature by up‐regulating the DAF‐9 sterol hormone signaling (Lee & Kenyon, 2009), while cool‐sensitive IL1 neurons promote lifespan at cool temperature through DAF‐16 in the intestine (Xiao et al., 2013; Zhang et al., 2018). Besides these direct effects of sensory neurons on longevity, neurons can also detect diverse cellular stressors and trigger animal‐wide stress responses to protect the animal and ensure survival (Durieux, Wolff, & Dillin, 2011; Prahlad, Cornelius, & Morimoto, 2008; Tatum et al., 2015; Taylor & Dillin, 2013). Therefore, different neuron types respond to distinct external and internal cues and activate distinct neuronal signals and signaling pathways to extend or shorten lifespan.

Consistent with the important role identified for the nervous system in aging, we find that the MT regulators appear to act in the nervous system to modulate longevity, as the lifespan phenotypes in MT regulator mutants are rescued by neuron‐specific transgenic expression of these MT regulators. However, MT regulators in the intestine might also have an effect on longevity, as intestinal expression of EFA‐6 can partially rescue the longevity phenotype in *efa‐6* mutants. It remains to be determined whether their function is required in all neurons or if a particular subset of neurons is more important for their effect on aging. Since the MT cytoskeleton plays critical structural role in cilia, and many genetic mutants that have impaired ciliary structures or functions display altered lifespan (Apfeld & Kenyon, 1999), it is possible that the MT regulators could regulate cilia function to affect lifespan. However, many of the long‐lived mutations affecting cilia lead to the disruption of ciliary structure, whereas we find that enhancing MT stability promotes instead of reduces lifespan. This could instead suggest a different mechanism such as alterations in the localization or release of vesicles encoding neurotransmitters or neuropeptides. More work will be needed to understand the mechanism(s) involved.

### 3.3 The role of MT regulators in regulating aging and metabolism requires DAF‐16

Insulin is involved in many cellular processes and the IIS pathway is known to play conserved roles in regulating metabolism, stress resistance, and lifespan (Kenyon, 2005). As one of the most important downstream transcription factors in IIS pathway, DAF‐16/FOXO regulates genes are involved in dauer formation, fat storage, stress response, and longevity (Murphy et al., 2003). IIS within the nervous system has been shown to be important for its role in longevity regulation in both *C. elegans* and mice (Iser et al., 2007; Taguchi et al., 2007; Wolkow et al., 2000). IIS pathway is involved in age‐dependent decay of neuron integrity. During aging, touch sensory neurons develop blebs and aberrant branches. These age‐dependent morphological changes are delayed in *daf‐2* mutants, and *daf‐16* is required for the beneficial effects in *daf‐2* mutants (Pan et al., 2011; Tank et al., 2011; Toth et al., 2012). We report that loss of EFA‐6 delays neuronal aging phenotypes in a DAF‐16‐dependent manner. We also show that MT regulators act in the nervous system to regulate lifespan, and that the lifespan extension in *efa‐6 *and *hdac‐6* mutants are dependent on DAF‐16. In addition, loss of DAF‐16 abolishes the effect on fat storage in *efa‐6* mutants, suggesting that the role of MT regulators in regulating metabolism and aging requires DAF‐16 function. Our RNA‐seq data showed that genes involved in metabolic pathways were differentially expressed in the MT regulator mutants. Many of these genes (e.g., fatty acid desaturase genes and acyl‐CoA dehydrogenase genes) are targets of DAF‐16 (Murphy et al., 2003). Among the genes whose expression is regulated in opposite directions between the two long‐lived and short‐lived MT regulator mutants, *nap‐1* encodes a conserved nucleosome assembly protein (NAP) family member that can function as a cofactor of DAF‐16 to regulate transcription of longevity genes (Cheong et al., 2013; Ihara et al., 2017). *acdh‐1* was previously reported as a target of DAF‐16 (Murphy et al., 2003). Also, *nap‐1* RNAi reduces *acdh‐1p::*GFP expression (MacNeil, Watson, Arda, Zhu, & Walhout, 2013), consistent with the notion that NAP‐1 might act as a cofactor of DAF‐16 to regulate transcription (Cheong et al., 2013). A previous study reported that *FoxO* regulates neuronal MT stability and its protein level was negatively regulated by MT disruption in *Drosophila* neurons (Nechipurenko & Broihier, 2012). In agreement with that, we detect increased DAF‐16 nuclear localization in *efa‐6* mutants. Together, these results suggest that neuronal MT status might affect DAF‐16 activity and subsequently affect fat metabolism and longevity. However, *daf‐18* RNAi, which increases insulin signaling (Gil et al., 1999; Mihaylova et al., 1999; Ogg & Ruvkun, 1998), displays an additive effect with *efa‐6(tm3124)* on longevity, suggesting that MT regulators might function in parallel with IIS pathway. Future study will be required to determine the precise mechanism by which DAF‐16 mediates the role of MT regulators in the context of aging.

## 4 EXPERIMENTAL PROCEDURES

### 4.1 *C. elegans* strains and transgenic animals

All *C. elegans *strains were maintained on standard NGM plates seeded with OP50 *E.coli* at 20 Celsius degree unless otherwise noted. The following *C. elegans *strains used in this study were obtained from the *C. elegans *Genetics Center which is funded by NIH Office of Research Infrastructure Programs (P40 OD010440): Bristol N2, CF702 *(muIs32(mec‐7p::GFP))*, RB2357 (*hdac‐6 (ok3203)*), RB809 (*ptl‐1(ok621)*), CF1038 (*daf‐16(mu86)*), PS3551 (*hsf‐1(sy441)*), QV225 (*skn‐1(zj15)*). *efa‐6(tm3124)* and *ptrn‐1(tm5597)* were obtained from the Mitani Laboratory. NM2689 (*jsIs821(mec‐7p::GFP::RAB‐3)*) was kindly provided by Dr. Michael Nonet (Bounoutas, Zheng, Nonet, & Chalfie, 2009). Double mutants were generated with standard genetic crosses, and the genotypes of strains were confirmed by PCR using primers detecting gene deletions or point mutations. Transgenic animals were generated by microinjection of 10 ng/μl plasmid DNA and 90 ng/μl Pttx‐3‐rfp plasmid as a co‐injection marker unless otherwise noted. At least two lines were tested for each transgene.

### 4.2 Fluorescence microscopy

*mec‐7p::GFP* was used to label touch neurons and *mec‐7p::GFP::RAB‐3 *was used to label synaptic vesicles in touch neurons. Well‐fed L4 animals were manually picked to synchronize the animals to regular NGM plates (40 worms/plate) and then transferred to NGM plates containing 50 μM FUDR (5‐fluoro‐2’‐deoxyuridine) on the first day of adulthood. Animals at the indicated days of adult life were mounted onto 2% agarose pad and immobilized with 30 μM muscimol (Sigma). Animals were then examined for neuronal morphology (blebbing and branching) or the distribution of GFP::RAB‐3 puncta using Zeiss LSM780 confocal microscope or Olympus IX83 inverted microscope. Each experiment was done in triplicate and results were compared to a control strain handled in parallel with the test strains. *muIs71* (DAF‐16::GFP) was used to examine DAF‐16 intracellular localization. Well‐fed L4 animals were manually picked and mounted, immobilized and scored/imaged using Olympus IX83 inverted microscope.

### 4.3 Touch sensitivity assay

Touch assay was performed as previously described (Chalfie & Sulston, 1981). Briefly, each animal was touched in the head and then tail with an eye bow hair for 5 times (10 touches in total). A positive response was determined as acceleration of the animal away from touch. The percentage of positive response was quantified.

### 4.4 Thrashing assay

All thrashing assays were done at room temperature (21.5–22.5°C). Thrashing assays were performed in 12‐well plates with 2 ml of M9 buffer per well. Individual worms were transferred to M9 buffer and allowed to recover for 20–30 s. Movies of 30 s were captured using IC Capture software with a DBK‐21BUC03 digital camera (ImagingSource, Germany) and a Zeiss Stemi 2000‐C Stereo Microscope. Movies were saved as AVI format and analyzed using ImageJ wrMTrck plugin (ImageJ1.48) for the number of body bends and the average speed. A body bend was defined as a change in the direction of the segment from the head to the midbody of an animal. The average speed was calculated as track length (mm)/time(s).

### 4.5 Lifespan assays

Animals were cultured at 20°C and survival was scored at room temperature (21.5–22.5°C). Well‐fed L4 animals were manually picked to synchronize the animals on regular NGM plates (~50 worms/plate × 2 plates) and these worms were transferred to NGM plates containing 50 μM FUDR on the first day of adulthood. If off springs were found in plates on Day 2, the adult animals would be transferred to another set of FUDR containing NGM plates. Animals were checked daily for dead or missing animals, and dead animals were removed from the plates when identified. The number of dead animals removed was recorded. Animals were considered dead when they failed to respond to touch and stopped pharyngeal pumping. RNAi treatment lifespans were conducted on NGM plates supplemented with FUDR, carbenicillin (50 μg/mL), isopropyl β‐d‐thiogalactopyranoside (IPTG, 1 mM). L4 animals were picked onto RNAi plates and the progenies at adult Day 1 stage will be picked to RNAi plates containing FUDR and tested for lifespan. Prism (Graphpad Software) was used to generate graphs and perform log‐rank testing for survival curve comparison. Lifespan data used to generate survival curves are summarized in Supporting information Table S2.

### 4.6 RNA‐seq

Wild‐type, *efa‐6(tm3124), hdac‐6(ok3203), ptl‐1(ok621),* and *ptrn‐1(tm5597)* worms were synchronized via hypochlorite treatment. When animals reached young adulthood, young adult animals were picked to fresh NGM plates with OP50 and allowed to lay eggs for 1 hr. The eggs were developed on regular NGM plates until L4 stage before transferred to plates containing FUDR. Adult Day 1 worms (three replicates for each strain) were collected for RNA extraction using Trizol followed by Qiagen RNeasy kit. 500 ng of total RNA from each sample was used to construct RNA‐seq libraries using KAPA RNA HyperPrep Kit with RiboErase (KK8483, KAPA Biosystems) following the manufacturer's protocol. Primers with barcoded adaptors (KK8710, KAPA Biosystems) were used to allow multiplex sequencing. Libraries were sequenced using Illumina HiSeq 3000 at the GCCRI Genome Sequencing Facility at UTHSCSA. The sequencing reads were aligned to *C. elegans* genome (ce10) using STAR 2.5.2b, and after removing reads mapped to rRNA sequences, read counts for each gene were conducted by featureCounts package with default parameters (Dobin et al., 2013; Liao, Smyth, & Shi, 2014). DESeq2 1.14.1 was used to call differentially expressed genes between each mutant and wild‐type groups, with more than 1.5‐fold expression change as the cutoff (Love, Huber, & Anders, 2014). The RNA‐seq data are available at the Gene Expression Omnibus under the accession number GSE115531 or the token "avqxsgogjxifvwv".

### 4.7 RT‐PCR

Synchronized adult Day 1 worms (four replicates for each strain) were collected for total RNA extraction using Trizol (Invitrogen). First‐strand cDNA was prepared from total RNA using the SuperScript III (Invitrogen). Real‐time PCR was performed with SyBR Green PCR Master Mix on CFX384 Real‐Time PCR Detection System (Bio‐Rad). Relative gene expression was analyzed with CFX Maestro software (Bio‐Rad). The following primers were used: 5′‐ acgtacagtccgacgagtcc ‐3′ (ama‐1‐F), 5′‐ agtacttggggctcgatgg ‐3′ (ama‐1‐R), 5′‐AGATAAGGAGGAAGACAAAC‐3′ (acdh‐1‐F), 5′‐CGTATTTGTAGCCTTTTCCA‐3′ (acdh‐1‐R), 5′‐GGGAGATCAACTCGCTGAATTG‐3′ (nap‐1‐F), 5′‐CATGCTCCTCGATGGCTTCA‐3′ (nap‐1‐R).

### 4.8 Fat staining

#### 4.8.1 Oil Red O staining

Staining was performed as previously described (Li et al., 2016). Briefly, well‐fed worms were washed with 1× PBS and fixed in 1% paraformaldehyde/PBS for 30 min with rocking. Samples were then flash frozen in liquid nitrogen followed by thawing in 37°C water bath for 3 total freeze/thaw cycles. Samples were washed three times with PBS and dehydrated with 60% isopropanol for 2 min and stained with 60% Oil Red O working solution. The Oil Red O working solution was prepared fresh by mixing 60% volume of Oil Red O stock with 40% volume of water. The Oil Red O stock solution was dissolved in isopropanol at a concentration of 0.5 g/100 ml and was equilibrated for several days prior to use. After staining, the samples were washed and mounted to 2% agarose padded slides for imaging using Zeiss Imager M2 microscope and Q‐IMAGING color camera (mbf Bioscience, Williston, VT). Intensity of Oil Red O staining was quantified using Image J.

#### 4.8.2 Nile Red staining

Staining was performed as previously described (Li et al., 2016). Similar to Oil Red O staining, well‐fed animals were fixed with 1% paraformaldehyde and treated with 3 freeze/thaw cycles and dehydrated in 60% isopropanol. Animals were then stained with 1 μg/ml Nile Red in 60% isopropanol for 30 min. Animals were washed and mounted to 2% agarose padded slides for imaging using Olympus IX83 microscope. Intensity of staining was quantified using Image J.

## CONFLICT OF INTEREST

None Declared.

## AUTHOR CONTRIBUTIONS

Conceived and designed the experiments: AX, AF, ZL, and LC. Performed the experiments: AX, SK, and LC. Analyzed the data: AX, ZZ, and LC. Wrote the paper: AX, ZZ, ZL, and LC.
