# Supplementary material for: Microtubule regulators act in the nervous system to modulate fat metabolism and longevity through DAF‐16 in C. elegans
Source: Aging Cell. 2019 Jan 14;18(2):e12884. doi: 10.1111/acel.12884 (PMC6413656; doi:10.1111/acel.12884)
Supplement: Supplementary file 1 [file ACEL-18-e12884-s001.pdf]

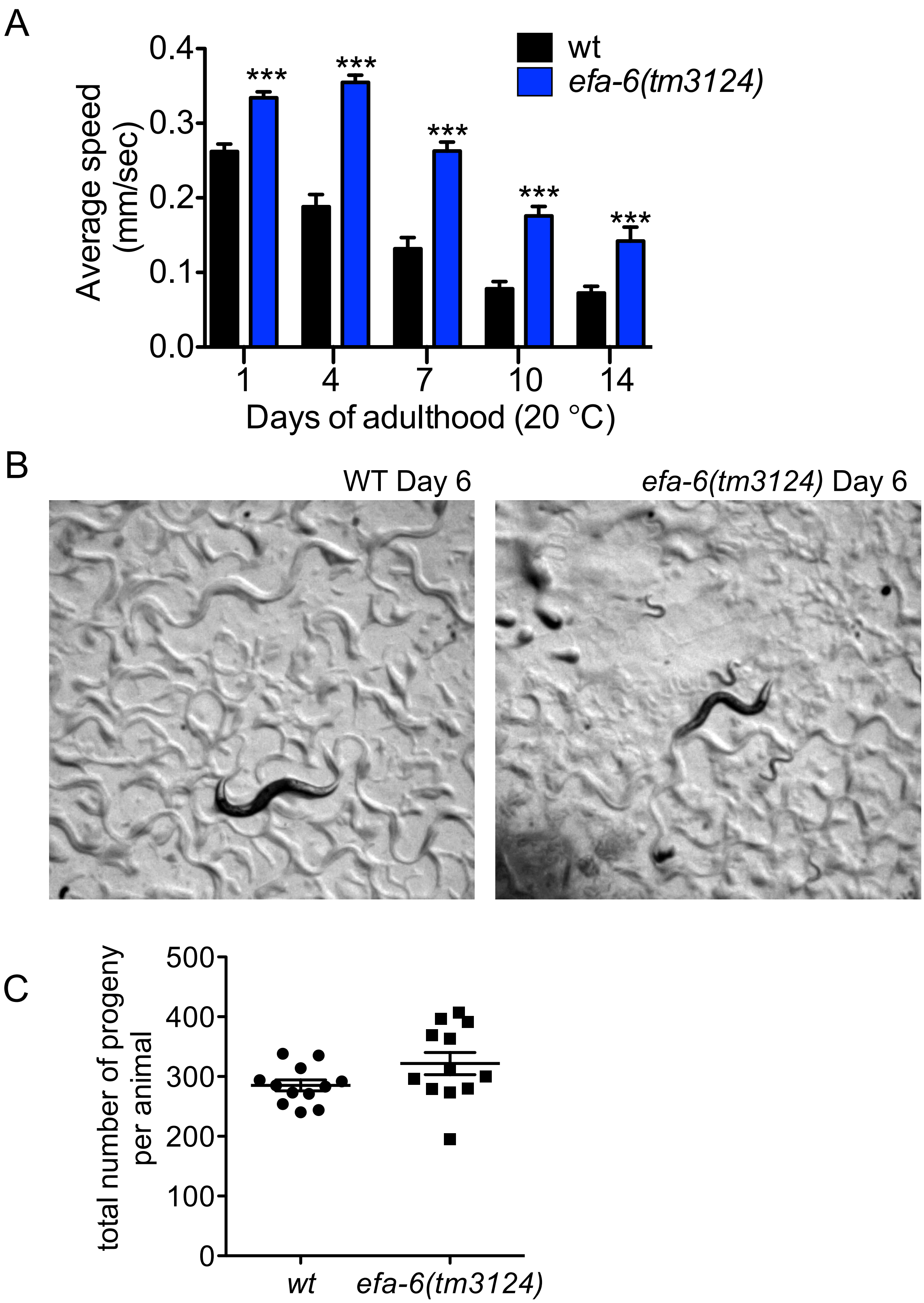

A
wt
efa-6(tm3124)
***
Average speed (mm/sec)
0.0
0.1
0.2
0.3
0.4
1
4
7
10
14
Days of adulthood (20 °C)
B
WT Day 6
efa-6(tm3124) Day 6
C
total number of progeny per animal
0
100
200
300
400
500
wt
efa-6(tm3124)

Figure S1

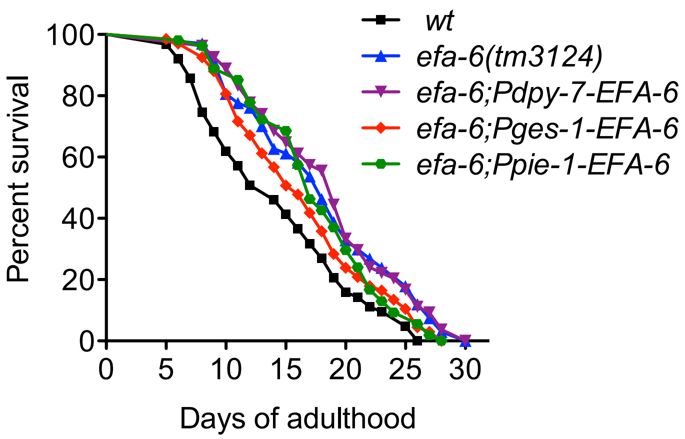

	Log-rank Test
efa-6 vs. efa-6;Pdpy-7-EFA-6	P = 0.7655
efa-6 vs. efa-6;Ppie-1-EFA-6	P = 0.2426
efa-6 vs. efa-6;Pges-1-EFA-6	P = 0.0794

Figure S2

A

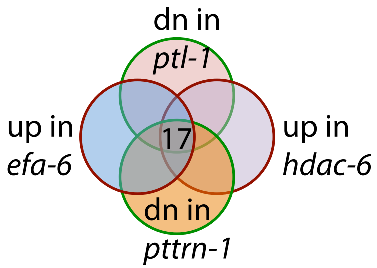

Gene	Locus
**acdh-1**	C55B7.4
C50F4.1	C50F4.1
col-103	F56B3.1
col-129	M18.1
col-142	T15B7.4
col-19	ZK1193.1
ifc-2	M6.1
K10C2.1	K10C2.1
mua-3	K08E5.3
**nap-1**	D2096.8
pab-1	Y106G6H.2
pcp-3	F23B2.11
pmp-5	T10H9.5
T05E12.6	T05E12.6
unc-54	F11C3.3
vab-10	ZK1151.1
Y69H2.14	Y69H2.14

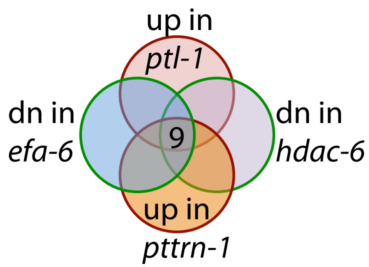

Gene	Locus
col-95	Y41C4A.16
F53H4.2	F53H4.2
his-1	T10C6.14
his-2	T10C6.13
his-3	T10C6.12
his-4	T10C6.11
his-7	F45F2.4
ttr-2	K03H1.4
Y53H1B.2	Y53H1B.2

B

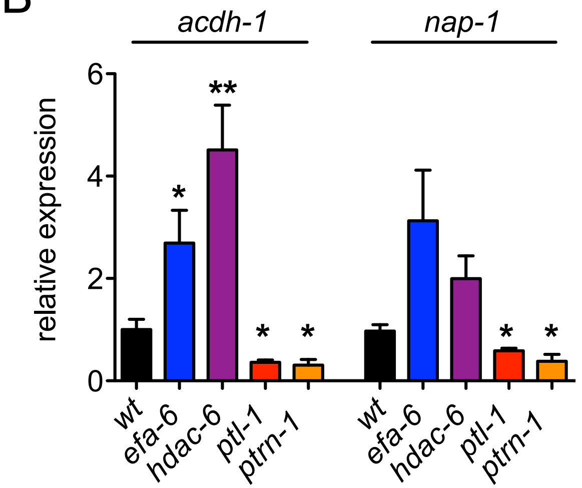

C

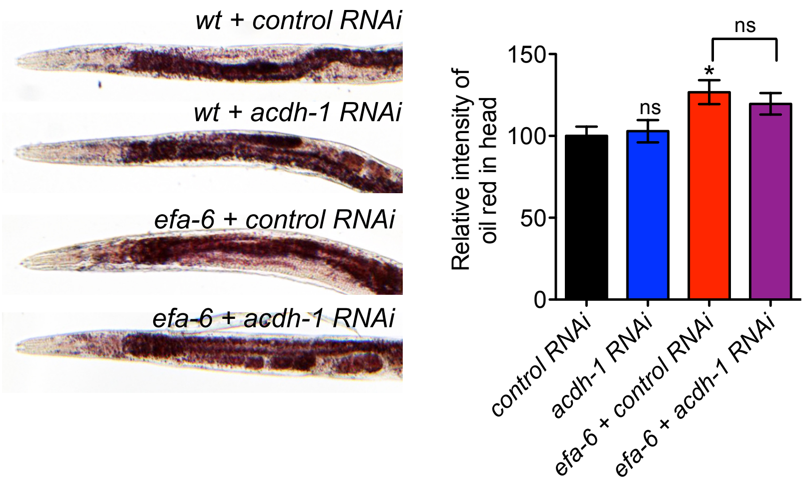

Figure S3
